# KDIGO 2024 clinical practice guideline on evaluation and management of chronic kidney disease: A primer on what pharmacists need to know

**DOI:** 10.1093/ajhp/zxaf044

**Published:** 2025-04-08

**Authors:** Linda Awdishu, Rebecca Maxson, Chelsea Gratt, Tamara Rubenzik, Marisa Battistella

**Affiliations:** Division of Clinical Pharmacy, University of California, San Diego Skaggs School of Pharmacy and Pharmaceutical Sciences, La Jolla, CA, USA; Harrison College of Pharmacy, Auburn University, Auburn, AL, USA; CVS Pharmacy, Inc., San Diego, CA, USA; Division of Nephrology, University of California, San Diego School of Medicine, La Jolla, CA, USA; Leslie Dan Faculty of Pharmacy, University of Toronto, Toronto, Canada, and University Health Network, Toronto, ON, Canada

**Keywords:** blood pressure, chronic kidney disease, diabetes, glomerular filtration rate, KDIGO practice guidelines, mineralocorticoid receptor antagonists, nephrotoxins, pharmacists, renin-angiotensin-aldosterone system

## Abstract

**Purpose:**

To review the key updates in the 2024 KDIGO clinical practice guideline for the evaluation and management of chronic kidney disease (CKD) and highlight the essential role of pharmacists in implementing these recommendations.

**Summary:**

The updated guideline introduces significant changes in CKD management, including the use of validated equations for estimating glomerular filtration rate (GFR) for drug dosing, with incorporation of serum cystatin C into GFR estimates for specific patient populations, and an emphasis on a comprehensive approach to delay disease progression. The guideline recommends sodium-glucose cotransporter 2 inhibitor (SGLT2i) therapy for kidney disease with proteinuria, with or without diabetes, renin-angiotensin-aldosterone system inhibitors (RAASi) blood pressure control and proteinuria management, and statins to reduce the risk of atherosclerotic cardiovascular disease. New evidence supports the use of finerenone in patients with type 2 diabetes and CKD, and GLP-1 receptor agonists for their kidney-protective effects. The guidelines also emphasize the importance of nephrotoxin stewardship and prevention of acute kidney injury through patient education on sick day medication management.

**Conclusion:**

Pharmacists play a crucial role in implementing these updated guidelines through comprehensive medication management, nephrotoxin stewardship, drug dosing adjustments, and patient education. Their involvement in interprofessional care teams is essential for optimizing health outcomes in patients with CKD.

In 2021, it was estimated that approximately 850 million individuals worldwide had chronic kidney disease (CKD).^1^ The care provided to individuals living with CKD is complex and multifaceted, incorporating early identification through screening, diagnostic testing, lifestyle and dietary modifications, novel therapeutics, and transitions of care to transplantation, dialysis, or end-of-life care. Given these complexities, the Kidney Diseases: Improving Global Outcomes (KDIGO) workgroup has developed guidance documents that incorporate evidence-based recommendations with expert-driven practice points for clinical management. In 2024, an update to the 2012 KDIGO clinical practice guideline (CPG) for the evaluation and management of CKD^2^ was published. The updated guideline includes refinement of kidney function estimation, population and individual risk prediction, and novel treatments demonstrated to have a significant impact on CKD prognosis.^1^ In this article, we will review the updates presented in the 2024 CPG,^1^ with a special focus on the role of the pharmacist in the care of patients with CKD and the perspective of a patient living with kidney disease. One of the authors (C.G.), a pharmacist practicing in community pharmacy, is living with CKD due to focal segmental glomerulosclerosis (FSGS). She has received multidisciplinary care (MDC) including pharmacist services and provides her unique experience as both a pharmacist and patient.

## Evaluation of CKD

The diagnosis of CKD is based upon abnormalities of kidney structure or function present for at least 3 months (Table 1). CKD is then classified based upon the cause of kidney disease, glomerular filtration rate (GFR) category, and degree of albuminuria (Tables 2 and 3).^1^

**Table 1.** Criteria for Chronic Kidney Disease Diagnosis

	Criteria
Markers of kidney damage^a^	Albuminuria (ACR ≥30 mg/g [in SI units, ≥3 mg/mmol]) Urine sediment abnormalities Persistent hematuria Electrolyte and other abnormalities due to tubular disorders Abnormalities on histology Structural abnormalities on imaging History of kidney transplantation
Decreased GFR^1^	GFR <60 mL/min per 1.73 m^2^

Abbreviations: ACR, albumin:creatinine ratio; GFR, glomerular filtration rate.

^a^Present for at least 3 months.

**Table 2.** Glomerular Filtration Rate Categories

eGFR category	eGFR, mL/min per 1.73 m^2^
G1	≥90
G2	60-89
G3a	45-59
G3b	30-44
G4	29-15
G5	<15

Abbreviation: eGFR, estimated glomerular filtration rate.

**Table 3.** Albuminuria Categories in Chronic Kidney Disease

Category	AER, mg/24 h	Urine ACR
A1	<30	<3 mg/g (<30 (mg/mmol)
A2	30-300	3-30 mg/g (30-300 (mg/mmol)
A3	>300	>30 mg/g (>300 (mg/mmol)

Abbreviations: ACR, albumin:creatinine ratio; AER, albumin excretion rate.

### Estimating GFR

The updated KDIGO guideline continues to recommend using serum creatinine (SCr) and an equation to estimate GFR.^1^ There are notable sources of error in determining estimated GFR (eGFR) using SCr, including non–steady-state conditions, non-GFR determinants of SCr, measurement errors at higher GFRs, and interference in laboratory assays. Non-GFR determinants of SCr include dietary intake of protein, extremes of muscle mass, and decreased creatinine secretion or extrarenal elimination due to medication use.^1^

**Key Points**

- Pharmacists can help delay chronic kidney disease progression by improving glycemic and blood pressure control by increasing use of RAASi, SGLT2i, GLP1RA, and finerenone.
- Pharmacists should be knowledgeable of patient conditions that may require alternative kidney function biomarkers to estimate glomerular filtration rate and adjust drug doses based on the best possible estimate.
- In addition to medication reconciliation and medication management, pharmacists should engage in nephrotoxin stewardship and educate patients on acute kidney injury prevention.

In situations where measuring eGFR using SCr might not be as accurate, an equation using both SCr and serum cystatin C (the eGFRcr-cysC equation) should be used, especially when it affects clinical decision-making such as drug dosing.^1^ This recommendation is supported in part by 2 large-scale studies of pooled cohorts in North America and Europe. The first was performed by the Chronic Kidney Disease Epidemiology Collaboration (CKD-EPI), and the second by the European Kidney Function Consortium (EKFC).^3,4^ Both groups developed eGFR equations whereby the P_30_ (defined as the percentage of eGFR values within ±30% of measured GFR) using the eGFRcr-cysC equation is in the range of 90%, which is considered optimal for an eGFR equation. In addition, both groups found greater accuracy for eGFR using a combined equation rather than the eGFRcr or eGFRcys equation alone. The sources of error and non-GFR determinants of serum cystatin C are less well known. These are thought to include obesity, inflammation, glucocorticoid excess, thyroid abnormalities, and smoking.^1^

The guidelines recommend the use of a validated eGFR equation rather than using serum filtration markers alone. Specifically, this includes the equations developed by CKD-EPI, the equations developed by EKFC, and validated modifications made by these equations for use in specific regions.^1^

### Race

Historically, the demographic variables of age, sex, and race were used in eGFR equations to explain variations in SCr that are unrelated to GFR, in an effort to obtain a more accurate estimate. However, it is now recognized that race is shaped by geographic, cultural, and sociopolitical forces and that the definition can change across geography and over time.^5^ In recent years, there has been increasing scrutiny over the use of race in eGFR equations around the world, which subsequently led to the 2021 recommendation that race no longer be used in the computation of eGFR in the United States.^6^ The 2021 CKD-EPI eGFRcr-cysC equation includes both filtration markers but does not have a term for race, and it is more accurate than the equations that use SCr or serum cystatin C alone.^3^ As a result, the combined equation is recommended when treatment decisions such as drug dosing are made based upon eGFR.

**A Patient’s Story**

*My journey with chronic kidney disease started 10 years ago when I first noticed swelling in my ankles. Initially, I attributed it to the long hours spent standing at my job. However, it quickly progressed to pitting edema, weight gain of around 30 pounds, and difficulty breathing. As I began the workup with my primary care doctor, functioning day to day became a huge challenge. My proteinuria had reached 10 g per day, and I was extremely uncomfortable. My ankles did not fit into any of my shoes, and the abdominal swelling forced me to wear maternity clothing. Maximum diuretic doses were not relieving the fluid overload, and I felt suffocated lying down. I would wake up in the middle of the night coughing and gasping for air, with my eyes almost swollen shut. It was truly a very scary time, and I was terrified that I might be in heart failure. After what felt like an eternity, a kidney biopsy confirmed my diagnosis of focal segmental glomerulosclerosis (FSGS). Essentially, I had scarring on some (but not all) of the glomeruli of my kidneys, and this was allowing protein that would normally stay in my blood to leak into my urine. Genetic testing for FSGS came back negative, and secondary conditions were ruled out. The cause was unclear, but there were some case reports suggesting infliximab, which I was receiving for another condition, as a potential cause of FSGS. Managing my chronic kidney disease has been both mentally and physically exhausting over the years. Luckily, my support system and amazing chronic kidney disease multidisciplinary team have helped me navigate this tricky disease. It’s tough not knowing if, and when, my disease will progress in the future, but at least for now, my proteinuria is stable and my glomerular filtration rate is preserved.*

**Patient perspective**

*Evaluating my chronic kidney disease from the patient perspective has changed a lot over the years. Initially, I fixated on my lab results, particularly my eGFR and ACR. I would let what would appear to be “worse” lab values affect my mood for days. As my focal segmental glomerulosclerosis has become more stable, I’ve learned to view my lab values with a more balanced perspective. Having appointments with my chronic kidney disease team and seeing my lab values every 4 months helps me stay motivated in terms of diet, exercise, and adherence. In between visits, I keep an eye on my blood pressures, swelling, and—although it’s subjective—how foamy my urine appears to be.*

### Albuminuria versus proteinuria

The KDIGO 2012 guidelines for the evaluation and management of CKD made the recommendation to focus on albuminuria rather than proteinuria, as albuminuria is the principal component of urinary protein in most kidney diseases.^2^ Even at low levels, there is a strong relationship between the quantity of urine albumin and the risk of kidney and cardiovascular disease (CVD) risk in addition to current CVD. Assays to measure urine albumin are more sensitive and precise than assays to measure urine protein. Initial testing for urine albumin should be done with a urine albumin:creatinine ratio (ACR) or a reagent strip urinalysis for albumin and ACR with automated reading. A positive reagent strip test should be confirmed with an ACR, and an abnormal ACR should be confirmed with subsequent first-morning void midstream urinalysis when possible.^1^

## Delaying CKD progression with evidence-based medicine

**Figure 1.**
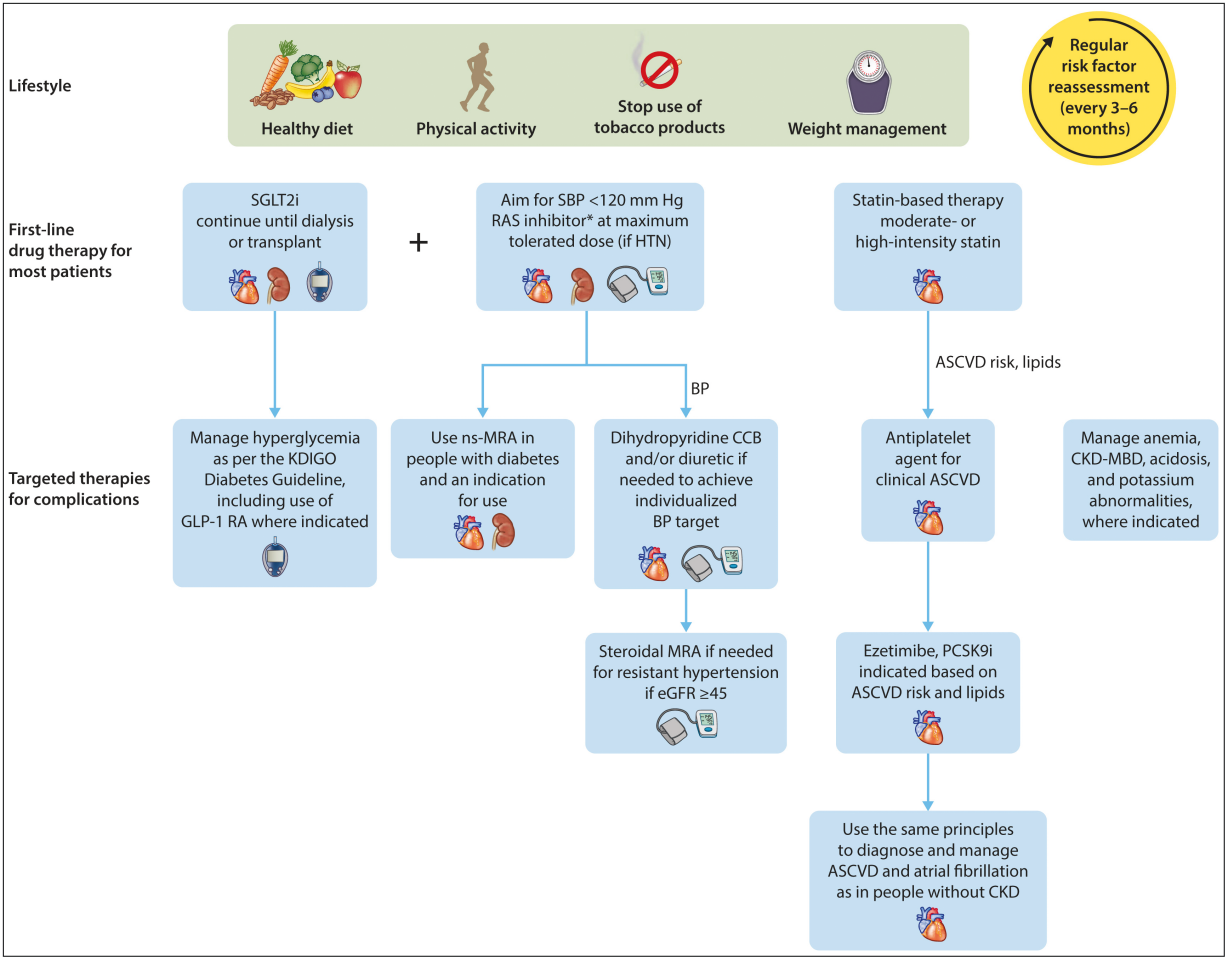
Holistic approach to chronic kidney disease (CKD) treatment and risk modification. Icons presented indicate the following benefits: blood pressure (BP) cuff = blood pressure lowering; glucometer = glucose-lowering; heart = heart protection; kidney = kidney protection; scale = weight management. ASCVD indicates atherosclerotic cardiovascular disease; CCB, calcium channel blocker; CKD-MBD, chronic kidney disease-mineral and bone disorder; eGFR, estimated glomerular filtration rate; GLP-1 RA, glucagon-like peptide 1 receptor agonist; HTN, hypertension; KDIGO, Kidney Disease: Improving Global Outcomes; MRA, mineralocorticoid receptor antagonist; ns-MRA, nonsteroidal mineralocorticoid receptor antagonist; PCSK9i, proprotein convertase subtilisin/kexin type 9 inhibitor; RAS, renin-angiotensin system; SBP, systolic blood pressure; SGLT2i, sodium-glucose cotransporter 2 inhibitor. This caption was adapted from reference 8. Illustration reprinted, with permission, from reference 1. *Angiotensin-converting enzyme inhibitor or angiotensin II receptor blocker should be first-line therapy for BP control when albuminuria is present; otherwise, dihydropyridine CCB or diuretic can also be considered. All 3 classes are often needed to attain BP targets.

Holistic management of individuals with CKD is emphasized in the latest KDIGO guidelines, with recognition of the interrelatedness of risk factors for CKD progression, CVD, and CKD complications (previously termed secondary complications of CKD).^1^ Recognition and treatment to address both CKD manifestations (eg, mineral bone disorder, anemia, reduced ability to complete activities of daily living, other clinical signs and symptoms) and CKD outcomes (eg, progression to kidney failure, CVD) are equally important.^1^ The 2024 KDIGO guideline made several nonpharmacological recommendations that are beyond the scope of this article but would be beneficial to review (see chapter 3, sections 3.2 and 3.3).^1^ The guideline also focuses on the use of medications proven to delay progression of CVD and/or improve CV morbidity and mortality. Three medication classes are considered first-line drug therapy for most patients and should be initiated when possible: sodium-glucose cotransporter 2 inhibitors (SGLT2i), renin-angiotensin-aldosterone system inhibitors (RAASi), and statin therapy. We refer the reader to a figure published with the guideline, reproduced with permission here (Figure 1).^1^

### Hypertension and diabetes goals

The updated guideline repeats the same blood pressure (BP) and glycated hemoglobin (HbA_1c_) goals specified in the KDIGO 2021 CPG for the management of BP in CKD^7^ and KDIGO 2022 CPG for diabetes management in CKD.^8^ The specific goals are shown in Table 4.

**Patient perspective**

*Delaying progression of my CKD started with prednisone and blood pressure control. There were a lot of medication adjustments as we tried to push maximum doses of lisinopril for proteinuria control in combination with diuretics for swelling. While initially I was only monitoring for high blood pressures, eventually I had to pay attention to when I was feeling dizzy and look out for low blood pressures as well. Ultimately, trials of prednisone and cyclosporine only elicited a partial response, and we had to move on to medications with less supporting evidence. We tried 3 more immunosuppressive medications, did a repeat kidney biopsy, and tried 2 types of apheresis, again with partial response. I was then incredibly fortunate to have 2 more options present themselves when it appeared we had exhausted all available options to explore. I started sparsentan in its phase 3 clinical trials and started dapagliflozin with approval of its new indication for kidney disease. We have had to rechallenge with dapagliflozin 3 times due to yeast infections (which are more related to my Crohn’s disease), but now I finally have the newest medicines on board and am taking them without issue.*

**Table 4.** Blood Pressure and Glycemic Goals for Slowing Progression of Chronic Kidney Disease

	Goal	Comments
Blood pressure^1,7^	SBP <120 mm Hg by standardized office BP measurement^9^ (<130 mm Hg if not standardized)	More patients will achieve SBP of <130 mm Hg if goal is <120 mm Hg Adjust goal for frailty, limited life expectancy, or history of falls or fractures Home-based monitoring can be beneficial
HbA_1c_ (CKD-ND)^8^	Individualized goal ranging from <6.5% to <8%	Factors to consider: CKD stage, macrovascular complications, comorbidities, life expectancy, hypoglycemia awareness, ability to manage hypoglycemia, and risk of hypoglycemia from treatment

CKD-ND, chronic kidney disease, non–dialysis dependent; SBP, systolic blood pressure; HbA_1c_, glycated hemoglobin.

#### RAASi

This medication class, including angiotensin-converting enzyme inhibitors (ACEi) and angiotensin receptor blockers (ARBs), remains essential in the treatment of CKD. The guideline authors again repeated recommendations from their recently published BP and diabetes guidelines.^7,8^ These are summarized in Box 1.

Box 1. **Summary of KDIGO Guideline Recommendations on Blood Pressure and Diabetes Management in CKD^7,8^**

- Use ACEi or ARB for people with CKD and moderately to severely increased albuminuria (these are level A2 and A3 recommendations):
  ◦ Level of evidence 1B for severely increased albuminuria without diabetes
  ◦ Level of evidence 2C for moderately increased albuminuria without diabetes
  ◦ Level of evidence 1B for moderately to severely increased albuminuria with diabetes
- Avoid combining ACEi, ARB, and direct renin inhibitor
- Use the highest approved and tolerated dose
- Check blood pressure, SCr, and serum potassium within 2 to 3 weeks of initiation or dose increase
- Treat hyperkalemia in preference to decreasing the dose of or stopping RAASi
- Consider reducing dose or stopping RAASi under the following circumstances:
  ◦ Symptomatic hypotension
  ◦ Uncontrolled hyperkalemia despite medical treatment
  ◦ To reduce uremic symptoms when eGFR is <15 mL/min/1.73m^2^

Abbreviations: ACEi, angiotensin-converting enzyme inhibitor; ARB, angiotensin receptor blocker; CKD, chronic kidney disease; eGFR, estimated glomerular filtration rate; KDIGO, Kidney Disease: Improving Global Outcomes; RAASi, renin-angiotensin-aldosterone system inhibitor; SCr, serum creatinine.

Two practice points added to the recommendations shown in Box 1 are pertinent to pharmacists. First, it is recommended that RAASi be started in individuals with CKD and normal to mildly increased ACR (category A1) to treat specific indications such as hypertension and heart failure with reduced ejection fraction (HFrEF).^1^ This recommendation assists in treatment of patients with overlapping comorbidities. An initial decrease in eGFR is expected when initiating a RAASi due to decreased intraglomerular pressure from vasodilation of the efferent arteriole.^9,10^ Of note, if the SCr increases by 30% or more from baseline, acute kidney injury (AKI) should be investigated. The workgroup recommends evaluating for and/or correcting volume depletion, concomitant medications (especially diuretics and nonsteroidal anti-inflammatory drugs [NSAIDs]), and considering renal artery stenosis.^1^ If this approach is ineffective at bringing the SCr back towards baseline, then a reduction in dose or discontinuation of RAASi is warranted.^1^ However, controversy exists over stopping RAASi; because patients are not often rechallenged with those agents, fewer patients receive these important disease-modifying treatments. The European Cardiology Society recommends continuing RAASi unless the SCr increases by more than 50% to improve utilization of goal-directed medical therapy in patients with HFrEF.^11^

Additionally, managing hyperkalemia with a view towards maintaining RAASi therapy is important, as retrospective studies have shown an increase in mortality and CV events in patients with CKD whose RAASi was discontinued for hyperkalemia.^12^ The suggested algorithm for managing hyperkalemia that occurs while on RAASi therapy along with key information on hyperkalemia (section 3.11 of the guideline^1^) are provided in Box 2. Treatment of hyperkalemia has improved over the past few years with the introduction of 2 new potassium exchange agents, patiromer and sodium zirconium cyclosilicate (See Table 5 for additional details). However, as they are brand-name agents, access can be difficult given that formulary coverage varies by region and by year and changes in availability to local pharmacies can occur. Patients should be rechallenged with a RAASi as soon as kidney function or hyperkalemia has been addressed to avoid high rates of discontinuation.^1^

Box 2. **Management of Hyperkalemia**

**Algorithm for managing hyperkalemia during RAASi therapy**

First line: Review concurrent drugs that cause hyperkalemia (discontinue if possible); assess dietary intake of potassium (reduce if possible).

Second line: Treat with diuretics, potassium exchange agents, and/or sodium bicarbonate (if renal tubular acidosis is suspected).

Third line: Reduce dose or discontinue RAASi; reassess at next scheduled CKD follow-up visit and restart in future if contributing factors have been addressed or resolved.

**Drugs associated with hyperkalemia**

- RAASi
- Aldosterone antagonists
- β-blockers
- Digoxin
- Heparin
- Potassium-sparing diuretics
- NSAIDs
- Calcinuerin inhibitors

Abbreviations: NSAID,nonsteroidal anti-inflammatory drug; RAASi, renin-angiotensin-aldosterone system inhibitor.

**Table 5.** Selected Information on Potassium Exchange Agents

	Polystyrene sulfonate sodium	Patiromer	Sodium zirconium cyclosilicate
Exchange action	Sodium for potassium	Calcium for potassium	Hydrogen and sodium ions exchanged for potassium
Binds in addition to potassium	Magnesium, calcium	Potassium, magnesium, phosphate	
Onset of effect	Variable, hours to days	4-7 hours	Within 1 hour
Duration of effect	Variable, 6-24 hours	24 hours	Not studied
Separate from other medications	3 hours before or after oral medications (6 hours in setting of gastroparesis)	3 hours before or after oral medications (eg, atorvastatin, dabigatran, furosemide, tacrolimus)	pH-dependent bioavailability: separate 2 hours before or after

The second new practice point considers how to manage RAASi use as eGFR declines to less than 30 mL/min/1.73m^2^. Based on the STOP-ACEi trial, neither stopping a RAASi nor initiating a RAASi conferred kidney or CV benefit.^13^ Thus, patients who are tolerating their RAASi (as evidenced by normokalemia and lack of disturbing uremic symptoms) may continue to take it. Conversely, there is no evidence to support initiation of a RAASi when the eGFR is less than 15 mL/min/1.73m^2^.

### SGLT2i

This was the first class of glucose-lowering therapies approved for management of type 2 diabetes mellitus (T2D) with demonstrated superiority to placebo with respect to CV outcomes in patients with T2D who have or are at risk for atherosclerotic cardiovascular disease (ASCVD).^14-16^ Interestingly, post hoc analyses of these studies demonstrated evidence of kidney protection, leading to dedicated kidney outcome trials such as the DAPA-CKD trial, which compared dapagliflozin 10 mg daily to placebo in patients with CKD with or without T2D and an eGFR of ≥25 to ≤75 mL/min/1.73 m^2^ and urine ACR of ≥200 to less than 5,000 mg/g.^17^ Both groups received ACEi or ARB therapy at maximum tolerated doses. Exclusion criteria included type 1 diabetes, polycystic kidney disease, lupus nephritis, antineutrophil cytoplasmic antibody vasculitis, and immunosuppressant treatment for kidney disease within 6 months before enrollment. The primary outcome was a composite of a ≥50% sustained decline in eGFR, onset of kidney failure, or death from CV or kidney causes. Compared to placebo, dapagliflozin significantly reduced occurrence of the primary outcome (9.2% vs 14.5%; hazard ratio [HR], 0.61; 95% CI, 0.51-0.72; *P* < 0.001), with a 31% relative risk reduction in death from any cause (HR, 0.69; 95% CI, 0.52-0.88; *P* = 0.004; number needed to treat, 48). The EMPA-KIDNEY trial compared empagliflozin 10 mg daily versus placebo in individuals with CKD, defined as an eGFR of 20-44 mL/min/1.73 m^2^ or eGFR of 45-89 mL/min/1.73 m^2^ with an ACR of ≥200 mg/g, who were prescribed a clinically appropriate dose of an ACEi or ARB.^18^ Exclusion criteria were consistent with those in the DAPA-CKD study. The primary composite outcome was kidney disease progression or CV death. Key secondary outcomes were hospitalization for heart failure or CV death, all-cause hospitalizations, and death from any cause. Compared to placebo, empagliflozin significantly reduced occurrence of the primary outcome (13.1% vs 16.9%; HR, 0.72; 95% CI, 0.64-0.82; *P* < 0.001), and there were fewer all-cause hospitalizations but no significant effect on hospitalization for heart failure or CV death or death from any cause.

The mechanisms by which SGLT2i exert their kidney protective effects remain incompletely understood. Systemic and glomerular hemodynamic changes, metabolic benefits, and attenuation of inflammatory and oxidative stress pathways are potential mechanisms through which SGLT2i alter the natural disease course of CKD.

#### Implications for practice

As per the KDIGO guideline,^1^ the following patient populations should be treated with an SGLT2i:

- Adults with T2D and CKD with an eGFR of ≥20 mL/min/1.73 m^2^
- Adults with CKD and HFrEF
- Adults with an eGFR of ≥20 mL/min/1.73 m^2^ and an ACR of ≥200 mg/g

While recommended, it is noteworthy that there is only moderate evidence for SGLT2i use in adults without T2D who have an eGFR of 20 to 44 mL/min/1.73m^2^ and an ACR less than 200 mg/g.^19^ An initial reduction in eGFR is expected with SGLT2i therapy. If tolerated, an SGLT2i should be continued until progression to dialysis initiation or kidney transplant.^1^ Routine monitoring of eGFR or electrolytes is recommended if there are concerns regarding volume status. No increased risk of hypoglycemia or urinary tract infections was observed. There is an increased risk of mild mycotic genital infections in men and women, which can be treated with topical antifungal agents. ^20^

Other trials are currently underway with the aim of exploring the effects of SGLT2i in kidney transplant recipients and patients with end-stage kidney disease (ESKD). The INFINITI trial (ClinicalTrials.gov identifier, NCT04965935)^21^ is a mechanistic trial enrolling kidney transplant patients with and without T2D, while the DAPA-HD trial (NCT05179668)^22^ aims to study CV benefits of dapagliflozin in patients on hemodialysis.

### Finerenone

Three published studies have demonstrated the effects of finerenone, a novel selective, nonsteroidal mineralocorticoid receptor antagonist, on CV and kidney outcomes in patients with T2D and CKD.^23-25^ Finerenone blocks the effects of aldosterone, a hormone that causes inflammation, fibrosis, and sodium retention in the heart and kidneys.^23,25^ Finerenone may be a safer alternative to the traditional steroid-based mineralocorticoid receptor antagonists, such as spironolactone and eplerenone, which have been associated with gynecomastia and hyperkalemia.^23^ Finerenone is more potent and selective for the mineralocorticoid receptor, potentially reducing these adverse effects.^26^

The FIDELIO-DKD trial randomized 5,734 patients with T2D and CKD who had a moderately increased ACR (30-300) along with an eGFR of 25-60 mL/min/1.73 m^2^ and a history of diabetic retinopathy or a severely increased ACR (300-5,000) and an eGFR of 25-75 mL/min/1.73 m^2^ to finerenone or placebo once daily in addition to maximally tolerated doses of an ACEi or ARB. Finerenone significantly reduced the occurrence of major adverse kidney outcomes by 18% compared with placebo.^23^ The FIGARO-DKD trial enrolled 7,437 patients with T2D and CKD who had an ACR of 30-300 and an eGFR of 25-90 mL/min/1.73 m^2^ or an ACR of 300-5,000 and an eGFR of at least 60 mL/min/1.73 m^2^. Finerenone significantly reduced the incidence of the primary outcome (a composite of death from CV causes, nonfatal myocardial infarction, nonfatal stroke, or hospitalization for heart failure) by 13% compared with placebo, mainly driven by a lower rate of hospitalization for heart failure.^24^

The Fidelity analysis pooled results from these studies and showed that finerenone significantly reduced the risk of a kidney composite outcome by 23% and the composite CV endpoint by 14% relative to placebo.^25^ No synergistic effect was observed between finerenone and either SGLT2i or glucagon-like peptide-1 receptor agonists (GLP1RA). No significant differences were observed in safety outcomes among both treatment arms, with the exception of hyperkalemia, which was more common with finerenone (14.0%) than with placebo (6.9%).^25^ The CONFIDENCE trial will evaluate whether SGLT2i plus finerenone is superior to either agent alone in reducing albuminuria in patients with T2D and CKD.^27^

#### Implications for practice

In both the FIDELIO-DKD and FIGARO-DKD studies,^23,24^ very few patients were taking an SGLT2i or GLP1RA, and in the patients taking both finerenone and an SGLT2i or a GLP1RA, no synergistic effects were observed. While these studies demonstrated benefit in terms of CV outcomes, we await the results of additional studies to demonstrate the effect of finerenone in heart failure (eg, the FINEARTS study). At this time, spironolactone should not be replaced with finerenone for HFrEF. The safety profile of finerenone was generally similar to that of placebo, with hyperkalemia being the most common adverse event and leading to permanent treatment discontinuation in a higher percentage of patients receiving finerenone versus placebo (in the FIDELIO trial, 2.3% vs 0.9%; in the FIGARO study, 1.2% vs 0.4%; and in the FIDELITY trial, 1.7% and 0.6%, respectively).^23-25^

The 3 studies provide strong evidence that finerenone, added to RAASi therapy, reduces the risk of CV and kidney events in patients with T2D and CKD who have a wide range of albuminuria levels and CKD stages. Finerenone may be a promising therapeutic option for this high-risk population, especially for those who have heart failure or are at high risk for developing it. However, the use of finerenone requires careful monitoring of serum potassium levels and eGFR, and dose adjustment or discontinuation may be necessary in some patients.^1^

### GLP1RA

GLP1RA are currently indicated for the management of diabetes and for weight loss. The KDIGO guideline recommends the use of GLP1RA, citing proven CV benefit in improving glycemic control inpatients with diabetes and CKD.^1^ The FLOW trial demonstrated that relative to placebo use, semaglutide 1 mg weekly was associated with a lower rate of major kidney adverse events in patients with T2D, CKD, and albuminuria.^28^ CKD was defined as an eGFR of 25 to <50 mL/min/1.73m^2^ and a urine ACR of 100-5,000 mg/g or an eGFR of 50-75 mL/min/1.73m^2^ with a urine ACR of 300-5,000 mg/g.^28^ Outcomes were evaluated based on SGLT2i use at baseline. Benefits of semaglutide in reducing kidney outcomes (total eGFR slope, major CV events, and all-cause death) were consistent in participants with/without baseline SGLT2i use.^29^ Based on these results, semaglutide should be used in patients with diabetes, CKD, and albuminuria.

## Medication management

### Stepwise versus pillar approach

Typical medication management of chronic conditions such as diabetes employs a stepwise approach in which drugs are sequentially added and then titrated or optimized based on efficacy or dose reduced/discontinued based on adverse effects.^30^ Each step involves evaluation of the drug outcomes before moving on to the addition of another drug.^30^ The pillar approach involves simultaneously starting all “pillar drugs” with evidence to support their use.^30^ For example, the 4-pillar approach to HFrEF involves simultaneously starting an angiotensin receptor/neprilysin inhibitor, SGLT2i, mineralocorticoid receptor antagonist, and β-blocker.^31^ In patients with T2D and CKD, a future pillar approach may involve simultaneously starting a RAASi, SGLT2i, GLP1RA, and finerenone.^30^ However, clinical trial study design for SGLT2i or finerenone research have required participants to be on an ACEi or ARB at trial entry.^18,23^ Clinical trials demonstrating the efficacy and safety of a pillar approach to medication management in CKD are lacking.

**Patient perspective**

*Preventing further kidney injury has always been a priority. After a few close calls and almost receiving ketorolac postoperatively, I now list NSAIDs as an allergy. Because my GFR doesn’t necessitate intervention with NSAIDs, I rely on my “allergy” as a precautionary measure. I’m also very mindful of my hydration status and have learned to balance medication adherence while intentionally skipping medication doses on some days to prevent further injury.*

### Nephrotoxin stewardship

Nephrotoxin stewardship involves the judicious use of nephrotoxins and dose adjustment of drugs for changes in kidney function to improve patient outcomes and safety through a reduction in adverse events, optimizing recovery from AKI, preventing progression to CKD, and reducing healthcare costs.^32^ Common medications listed in the new KDIGO guideline include NSAIDs, proton pump inhibitors, herbal supplements, and antimicrobials.^1^ Evaluation of risk/benefit for each nephrotoxin is warranted, and some may need to be continued with careful monitoring of kidney function. Where possible, alternative medications should be used when the benefit from continuing the nephrotoxin is unclear (eg, substituting histamine-2 receptor antagonists for proton pump inhibitors in the setting of gastrointestinal reflux with no documented ulcer or serious hypersecretory state).

### Drug dose adjustment for kidney function

One of the most important changes in the new KDIGO guideline is a recommendation to dose drugs using a validated eGFR equation and SCr.^1^ This recommendation is based on several advantages of the latest eGFR equations, including large samples more representative of patients with and without kidney disease, standardization of the assays for SCr, and incorporation of additional kidney function biomarkers, namely serum cystatin C.^1^ This recommendation is a big change, moving away from using the Cockcroft-Gault equation to estimate creatinine clearance and adjust drug doses to using eGFR and, in some circumstances, serum cystatin C–based eGFR values. In light of this recommendation, the National Kidney Foundation (NKF) convened a pharmacy task force to develop recommendations on the use of eGFR for the purposes of drug dosing, and those recommendations are currently under review. The task force has endorsed the KDIGO recommendation to use eGFR based on validated race-free equations and to use SCr or serum cystatin C or a combination of biomarkers.^1^ Current validated equations provide standardized eGFR values adjusted to a body surface area (BSA) of 1.73m^2^. This approach is useful for population-based surveillance and identification of CKD across patient populations. However, for the purposes of drug dosing, KDIGO and the NKF pharmacy task force recommend BSA-adjusted eGFR should be used (this recommendation is under review).^1^ The BSA-adjusted value can be calculated by multiplying the standardized eGFR results by the person’s BSA and dividing by 1.73 m^2^ (eg, a patient with an eGFR of 40 mL/min/1.73m^2^ and BSA of 1.5 m^2^ has a BSA-adjusted eGFR of 35 mL/min) or by using the NKF eGFR calculator (https://www.kidney.org/professionals/kdoqi/gfr_calculator). Currently, the Food and Drug Administration guidance on pharmacokinetic studies suggests manufacturers may employ any contemporary equation for kidney function estimation, but when using eGFR, the BSA-adjusted value should be utilized.^33^ Consequently, drugs may be studied using different equations. The BSA-adjusted eGFR can be used for dosing most drugs. In situations where the SCr may not be reliable, the addition of serum cystatin C to the eGFR equation improves the precision of the values, and the combination biomarker equation may be used for drug dosing.^1^ These patient situations are defined in the guideline (eg, frail patients or patients with liver failure).^1^ However, all of the eGFR equations are for use in patients with steady-state kidney function. In the setting of AKI, a non–steady-state equation should be employed.^34^ The key point for clinicians is to determine the best possible estimate of kidney function and employ a risk/benefit approach to drug dosing (eg, conservative dosing for narrow therapeutic index drugs or aggressive dosing for high-risk clinical conditions). Where feasible, therapeutic drug monitoring should be employed for narrow therapeutic index drugs.

### Prevention of AKI

Sick day medication guidance (SDMG) is an important educational initiative to reduce the risk of AKI and other adverse events when patients are acutely ill and dehydrated. SDMG is a set of recommendations for withholding or adjusting specific medications in the setting of acute dehydrating illness that could lead to hypotension, hypoglycemia, AKI, or diabetic ketoacidosis.^35^ Recommendations include withholding SGLT2i, ACEi, diuretics (loop, thiazide, and potassium-sparing), metformin, ARBs, and NSAIDs, which is represented by the acronym SADMANs.

## Pharmacist role

With an emphasis on improving health equity along with morbidity and mortality, the new guidelines also focus on MDC that includes pharmacists.^1^ Throughout the guideline, clinical pharmacists are recommended as a resource for improving the lives of individuals with CKD in the following ways:

- Implementation of new eGFR equations, especially within electronic health records
- Assistance with lifestyle modifications (exercise, diet, and smoking cessation)
- Providing comprehensive medication management
- Improving pharmacoequity
- Reducing polypharmacy and improving drug stewardship

All these activities can be provided by pharmacists practicing in dialysis centers, outpatient nephrology practices, and inpatient settings. Awdishu and colleagues^36,37^ published their experience achieving Joint Commission certification in CKD by providing MDC for patients with stage 2 to 5 CKD. They demonstrated improvement in various quality indicators (eg, BP control, medication adherence, education on SDMG, and NSAID avoidance). Dyer and Awdishu^38^ published results of a study evaluating medication reconciliation in patients on chronic dialysis and the development of a novel “med to chair” program. This program included delivery of medication refills to the patient during the routine dialysis session along with counseling from the dialysis pharmacist. Including pharmacists in all settings ensures that all individuals with CKD receive the benefits of pharmacists’ interventions.

**Pharmacists’ role: education and counseling**

Asking a combination of both open-ended and targeted questions will help assess symptoms, adherence, hesitations, and potential issues that require further evaluation. Gathering perceptions and thoughts from a patient first will help to evaluate health literacy level and determine the best way to tailor your approach (Is the patient a visual thinker? Is there a language barrier, etc). A different approach may be needed if the person being education is a medical professional. Some patients will be happy to be counseled that their ACEi/ARB is kidney protective, while others will appreciate a more detailed explanation and have follow-up questions. Pharmacists in this setting can also take a more comprehensive approach in regard to overall health and lifestyle factors (eg, reviewing home BP readings, when to skip certain medications, salt intake, hydration/electrolytes).

**Pharmacists’ role**

Pharmacists working in ambulatory care or community settings are in an ideal position to support patients with CKD between visits. Despite limited access to patient information compared to other healthcare settings, pharmacists should be utilizing their knowledge and available information to take a proactive approach. Medication reconciliation in these settings involves using available diagnosis codes, inferring information from a patient’s profile, and asking the patient follow-up questions to determine if a prescription is safe for the patient or warrants a call to the physician. The most common interventions involve nephrotoxic combinations and counseling on dose changes or replacements. It is important to keep a patient’s profile up-to-date and inactivate old therapies to prevent automatic refills. Pharmacists should intervene at any point when a prescription is dispensed and counsel for comprehension to avoid duplicate or old therapies from being continued. A simple printout of current medications and the recommendation of a pill box can make a huge difference in simplifying a complex regimen for a patient with CKD.

OTC medication recommendations: One of the most common questions is “What can I take for pain?” Before suggesting an NSAID, consider the patient’s kidney risk. Asking follow-up questions (eg, “Do you have any health conditions (diabetes, hypertension, etc)? Are you on ACEi/ARB, diuretic, or SGLT2i?”) will help determine if an NSAID truly is the best option.

Provider access: Outpatient pharmacists can effectively mitigate delays in therapy and are well versed on medication formularies and finding solutions for drugs that are not covered. If a prior authorization or alternative is required, it should be immediately communicated to the prescriber so the process can move forward as quickly as possible. Outpatient pharmacists also connect patients with other ways of saving, such as drug manufacturer savings programs and discount cards. When a prior authorization is the only option available, outpatient pharmacists should explain the process in detail to patients, helping them understand the steps that need to be taken for the medication to be covered.

Much has been published from primary care settings regarding the benefits from MDC that includes a pharmacist providing comprehensive medication management (CMM). These benefits include improved clinical outcomes, fewer follow-up visits with the primary care provider, decreased costs, and decreased readmissions.^39-42^ Outcome data on pharmacoequity benefits of CMM pharmacists, including the impact on social determinants of health (SDOH), is limited. As a step towards this data, Petska and colleagues^43^ demonstrated that SDOH can be incorporated into CMM visits. Structured interviews with clinical pharmacists currently providing CMM in various nephrology settings showed that barriers to fully implementing CMM in the nephrology space remain.^44^ The Advancing Kidney Health through Optimal Medication Management (AKHOMM) initiative is currently working towards advancing pharmacy practice into nephrology care teams (see commentary article by Meaney and colleagues in this issue).

## Conclusion

The 2024 CPG on the evaluation and management of CKD includes important updates on determing eGFR using SCr and SCysC, delaying CKD progression by improving glycemic and BP control, increasing use of RAASi, SGLT2i, GLP1RA, and finerenone.^1^ Pharmacists can play an important role in the care of patients living with CKD through medication reconciliation, CMM, nephrotoxin stewardship, prevention of AKI through patient education and empowerment, and adjusting the doses of drugs eliminated by the kidney.^1^

## Data Availability

No new data were generated or analyzed in support of this article.

## Notes

**This article is part of a special *AJHP* theme issue on management of chronic kidney disease. Contributions to this issue were coordinated by Wendy L. St. Peter, PharmD, FCCP, FASN, FNKF; Rebecca A. Maxson, PharmD, BCPS; and Katie E. Cardone, PharmD, BCACP, FNKF, FASN, FCCP.**

## References

[1] Kidney Disease: Improving Global Outcomes CKD Work Group. KDIGO 2024 clinical practice guideline for the evaluation and management of chronic kidney disease. Kidney Int. 2024;105(4S):S117-S314. doi: https://doi.org/10.1016/j.kint.2023.10.018 38490803

[2] Stevens PE , Levin A; Kidney Disease: Improving Global Outcomes Chronic Kidney Disease Guideline Development Work Group M. Evaluation and management of chronic kidney disease: synopsis of the Kidney Disease: Improving Global Outcomes 2012 clinical practice guideline. Ann Intern Med. 2013;158(11):825-30. doi: https://doi.org/10.7326/0003-4819-158-11-201306040-00007 23732715

[3] Inker LA , Eneanya ND, Coresh J, et al New creatinine- and cystatin c-based equations to estimate GFR without race. N Engl J Med. 2021;385(19):1737-1749. doi: https://doi.org/10.1056/NEJMoa2102953 34554658 PMC8822996

[4] Pottel H , Bjork J, Rule AD, et al Cystatin C-based equation to estimate GFR without the inclusion of race and sex. N Engl J Med. 2023;388(4):333-343. doi: https://doi.org/10.1056/NEJMoa2203769 36720134

[5] Borrell LN , Elhawary JR, Fuentes-Afflick E, et al Race and genetic ancestry in medicine — a time for reckoning with racism. N Engl J Med. 2021;384(5):474-480. doi: https://doi.org/10.1056/NEJMms2029562 33406325 PMC8979367

[6] Delgado C , Baweja M, Burrows NR, et al Reassessing the inclusion of race in diagnosing kidney diseases: an interim report from the NKF-ASN task force. Am J Kidney Dis. 2021;78(1):103-115. doi: https://doi.org/10.1053/j.ajkd.2021.03.008 33845065 PMC8238889

[7] Kidney Disease: Improving Global Outcomes Blood Pressure Work G. KDIGO 2021 clinical practice guideline for the management of blood pressure in chronic kidney disease. Kidney Int. 2021;99(3S):S1-S87. doi: https://doi.org/10.1016/j.kint.2020.11.003 33637192

[8] Kidney Disease: Improving Global Outcomes Diabetes Work G. KDIGO 2022 clinical practice guideline for diabetes management in chronic kidney disease. Kidney Int. 2022;102(5S):S1-S127. doi: https://doi.org/10.1016/j.kint.2022.06.008 36272764

[9] Bjorck S , Mulec H, Johnsen SA, Norden G, Aurell M. Renal protective effect of enalapril in diabetic nephropathy. BMJ. 1992;304(6823):339-343. doi: https://doi.org/10.1136/bmj.304.6823.339 1540729 PMC1881212

[10] Clase CM , Barzilay J, Gao P, et al Acute change in glomerular filtration rate with inhibition of the renin-angiotensin system does not predict subsequent renal and cardiovascular outcomes. Kidney Int. 2017;91(3):683-690. doi: https://doi.org/10.1016/j.kint.2016.09.038 27927602

[11] Mullens W , Martens P, Testani JM, et al Renal effects of guideline-directed medical therapies in heart failure: a consensus document from the Heart Failure Association of the European Society of Cardiology. Eur J Heart Fail. 2022;24(4):603-619. doi: https://doi.org/10.1002/ejhf.2471 35239201

[12] Leon SJ , Whitlock R, Rigatto C, et al Hyperkalemia-related discontinuation of renin-angiotensin-aldosterone system inhibitors and clinical outcomes in ckd: a population-based cohort study. Am J Kidney Dis. 2022;80(2):164-173 e1. doi: https://doi.org/10.1053/j.ajkd.2022.01.002 35085685

[13] Bhandari S , Mehta S, Khwaja A, et al Renin-angiotensin system inhibition in advanced chronic kidney disease. N Engl J Med. 2022;387(22):2021-2032. doi: https://doi.org/10.1056/NEJMoa2210639 36326117

[14] Neal B , Perkovic V, Mahaffey KW, et al Canagliflozin and cardiovascular and renal events in type 2 diabetes. N Engl J Med. 2017;377(7):644-657. doi: https://doi.org/10.1056/NEJMoa1611925 28605608

[15] Wiviott SD , Raz I, Bonaca MP, et al Dapagliflozin and cardiovascular outcomes in type 2 diabetes. N Engl J Med. 2019;380(4):347-357. doi: https://doi.org/10.1056/NEJMoa1812389 30415602

[16] Zinman B , Wanner C, Lachin JM, et al Empagliflozin, cardiovascular outcomes, and mortality in type 2 diabetes. N Engl J Med. 2015;373(22):2117-2128. doi: https://doi.org/10.1056/NEJMoa1504720 26378978

[17] Heerspink HJL , Stefansson BV, Correa-Rotter R, et al Dapagliflozin in patients with chronic kidney disease. N Engl J Med. 2020;383(15):1436-1446. doi: https://doi.org/10.1056/NEJMoa2024816 32970396

[18] The E-KCG , Herrington WG, Staplin N, et al Empagliflozin in patients with chronic kidney disease. N Engl J Med. 2023;388(2):117-127. doi: https://doi.org/10.1056/NEJMoa2204233 36331190 PMC7614055

[19] Cherney DZI , Dekkers CCJ, Barbour SJ, et al Effects of the SGLT2 inhibitor dapagliflozin on proteinuria in non-diabetic patients with chronic kidney disease (DIAMOND): a randomised, double-blind, crossover trial. Lancet Diabetes Endocrinol. 2020;8(7):582-593. doi: https://doi.org/10.1016/S2213-8587(20)30162-5 32559474

[20] Lega IC , Bronskill SE, Campitelli MA, et al Sodium glucose cotransporter 2 inhibitors and risk of genital mycotic and urinary tract infection: a population-based study of older women and men with diabetes. Diabetes Obes Metab. 2019;21(11):2394-2404. doi: https://doi.org/10.1111/dom.13820 31264755

[21] University Health Network, Toronto. Efficacy, mechanisms and safety of SGLT2 inhibitors in kidney transplant recipients (INFINITI2019). In: ClinicalTrials.gov. https://www.clinicaltrials.gov/study/NCT04965935?term=NCT04965935

[22] Medical University of Vienna. SGLT2 inhibition in hemodialysis (DAPA-HD). In: ClinicalTrials.gov. https://clinicaltrials.gov/study/NCT05179668

[23] Bakris GL , Agarwal R, Anker SD, et al Effect of finerenone on chronic kidney disease outcomes in type 2 diabetes. N Engl J Med. 2020;383(23):2219-2229. doi: https://doi.org/10.1056/NEJMoa2025845 33264825

[24] Pitt B , Filippatos G, Agarwal R, et al Cardiovascular events with finerenone in kidney disease and type 2 diabetes. N Engl J Med. 2021;385(24):2252-2263. doi: https://doi.org/10.1056/NEJMoa2110956 34449181

[25] Agarwal R , Filippatos G, Pitt B, et al Cardiovascular and kidney outcomes with finerenone in patients with type 2 diabetes and chronic kidney disease: the FIDELITY pooled analysis. Eur Heart J. 2022;43(6):474-484. doi: https://doi.org/10.1093/eurheartj/ehab777 35023547 PMC8830527

[26] Rico-Mesa JS , White A, Ahmadian-Tehrani A, Anderson AS. Mineralocorticoid receptor antagonists: a comprehensive review of finerenone. Curr Cardiol Rep. 2020;22(11):140. doi: https://doi.org/10.1007/s11886-020-01399-7 32910349

[27] Green JB , Mottl AK, Bakris G, et al Design of the COmbinatioN effect of FInerenone anD EmpaglifloziN in participants with chronic kidney disease and type 2 diabetes using a UACR Endpoint study (CONFIDENCE). Nephrol Dial Transplant. 2023;38(4):894-903. doi: https://doi.org/10.1093/ndt/gfac198 35700142 PMC10064838

[28] Perkovic V , Tuttle KR, Rossing P, et al Effects of semaglutide on chronic kidney disease in patients with type 2 diabetes. N Engl J Med. 2024;391(2):109-121. doi: https://doi.org/10.1056/NEJMoa2403347 38785209

[29] Mann JFE , Rossing P, Bakris G, et al Effects of semaglutide with and without concomitant SGLT2 inhibitor use in participants with type 2 diabetes and chronic kidney disease in the FLOW trial. Nat Med. 2024;doi: https://doi.org/10.1038/s41591-024-03133-0 PMC11485243 38914124

[30] Morales J , Dagogo-Jack S, Fonseca V, Neumiller JJ, Rosas SE. Perspectives on chronic kidney disease with type 2 diabetes and risk management: practical viewpoints and a paradigm shift using a pillar approach. Clin Diabetes. 2023;41(4):553-566. doi: https://doi.org/10.2337/cd22-0110 37849516 PMC10577512

[31] Straw S , McGinlay M, Witte KK. Four pillars of heart failure: contemporary pharmacological therapy for heart failure with reduced ejection fraction. Open Heart. 2021;8(1)doi: https://doi.org/10.1136/openhrt-2021-001585 PMC7929859 33653703

[32] Kane-Gill SL. Nephrotoxin stewardship. Crit Care Clin. 2021;37(2):303-320. doi: https://doi.org/10.1016/j.ccc.2020.11.002 33752857

[33] Administration FaD. Pharmacokinetics in patients with impaired renal function – study design, data analysis, and impact on dosing guidance for industry. https://www.fda.gov/media/78573/download

[34] Awdishu L , Connor AI, Bouchard J, Macedo E, Chertow GM, Mehta RL. Use of estimating equations for dosing antimicrobials in patients with acute kidney injury not receiving renal replacement therapy. J Clin Med. 2018;7(8)doi: https://doi.org/10.3390/jcm7080211 PMC6111623 30103503

[35] Watson KE , Dhaliwal K, McMurtry E, et al Sick day medication guidance for people with diabetes, kidney disease, or cardiovascular disease: a systematic scoping review. Kidney Med. 2022;4(9):100491. doi: https://doi.org/10.1016/j.xkme.2022.100491 36046611 PMC9420951

[36] Awdishu L , Moore T, Morrison M, Turner C, Trzebinska D. A primer on quality assurance and performance improvement for interprofessional chronic kidney disease care: a path to Joint Commission certification. Pharmacy (Basel). 2019;7(3)doi: https://doi.org/10.3390/pharmacy7030083 PMC6789732 31277293

[37] Awdishu L , Singh RF, Saunders I, et al Advancing pharmacist collaborative care within academic health systems. Pharmacy (Basel). 2019;7(4)doi: https://doi.org/10.3390/pharmacy7040142 PMC6958419 31614555

[38] Dyer SA , Nguyen V, Rafie S, Awdishu L. Impact of medication reconciliation by a dialysis pharmacist. Kidney360. 2022;3(5):922-925. doi: https://doi.org/10.34067/KID.0007182021 36128498 PMC9438412

[39] McFarland MS , Buck ML, Crannage E, et al Assessing the impact of comprehensive medication management on achievement of the quadruple aim. Am J Med. 2021;134(4):456-461. doi: https://doi.org/10.1016/j.amjmed.2020.12.008 33472055

[40] Isetts BJ , Schondelmeyer SW, Artz MB, et al Clinical and economic outcomes of medication therapy management services: the Minnesota experience. J Am Pharm Assoc (2003). 2008;48(2):203-214. doi: https://doi.org/10.1331/JAPhA.2008.07108 18359733

[41] Brajkovic A , Bosnar L, Nascimento M, et al Healthcare utilisation and clinical outcomes in older cardiovascular patients receiving comprehensive medication management services: a nonrandomised clinical study. Int J Environ Res Public Health. 2022;19(5)doi: https://doi.org/10.3390/ijerph19052781 PMC8910212 35270472

[42] Mcfarland MS NJ , Ourth H, Groppi J, Morreale A. Optimizing the primary care clinical pharmacy specialist: increasing patient access and quality of care within the Veterans Health Administration. J Am Coll Clin Pharm. 2020;3(2):494-500.

[43] Pestka DL EC , Sorge LA, Funk KA. Incorporating social determinants of health into comprehensive medication management: insights from the field. J Am Coll Clin Pharm. 2020;3(6):1038-1047.

[44] Pestka DL MR , Bradley S, Hsu Y, Traynor A, St. Peter WL. Determining best practices to delivering comprehensive medication management in practices with patients with chronic kidney disease: a qualitative study. J Am Coll Clin Pharm 2022;5(11):1121-1127.

